# Unnecessary Needling: A Case of Iatrogenic Pneumothorax Following Dry Needling Procedure for Chronic Myofascial Pain

**DOI:** 10.7759/cureus.76055

**Published:** 2024-12-20

**Authors:** Alok Arora, Yuvraj Arora, Sanjana Vasireddy

**Affiliations:** 1 Internal Medicine, Aurora Medical Centre-Bay Area, Marinette, USA; 2 Pre-Medical, Homestead High School, Mequon, USA

**Keywords:** accupuncture, dry needling, interventional pain managem, physical therapy rehabilitation, secondary pneumothorax

## Abstract

This case report highlights a complication of pneumothorax associated with dry needling (DN), a technique used for the treatment of myofascial pain syndrome and musculoskeletal disorders. Despite its growing popularity and efficacy in relieving pain, dry needling can lead to adverse events. We present a case of a 35-year-old female who developed pneumothorax following a dry needling session. During the dry needling session, the patient reported sharp pain underneath the scapula, and pneumothorax was confirmed three days later on a visit to the emergency department. Even though the chances of a pneumothorax are slim when DN is conducted over lung fields, it is essential that patients are informed of potential risks, including chest pain/tightness, fatigue, or shortness of breath during the procedure.

## Introduction

Dry needling (DN), also known as intramuscular stimulation, is a therapeutic technique that involves inserting thin filiform needles into trigger points to alleviate muscle pain and dysfunction [1]. This practice has its roots in traditional acupuncture but has evolved significantly with advances in medical research and clinical practice. This technique is increasingly utilized in pain management and musculoskeletal rehabilitation due to its efficacy in treating chronic and acute pain conditions. 

While considered safe, dry needling carries risks, including infection, bleeding, and in extremely rare cases, more severe complications like pneumothorax, a condition in which air escapes from the lung to fill the space between the lung and chest wall [2]. As the demand for dry needling increases, driven by its success in both pain management and musculoskeletal rehabilitation, it is essential for both medical practitioners and prospective patients to be completely aware of the potential risks involved with the process [3]. Pain may occur at any given point during the dry needling session, and patients should be advised in advance to help prevent any discomfort. 

Clinical presentation of pneumothorax varies depending on the type of pneumothorax and the extent of air in the pleural space. Patients may describe immediate symptoms at the time the pneumothorax starts to develop, or the symptoms may be delayed, particularly in younger and otherwise healthy patients who can tolerate the main physiologic consequences of a decrease in vital capacity and partial pressure of oxygen fairly well, with minimal changes in vital signs and symptoms as noted in this case.

This case report discusses the clinical presentation and management of pneumothorax that happened during DN in the periscapular region. Along with that, this case report underscores the importance of recognizing and addressing complications associated with dry needling, ensuring both patient safety and therapeutic success. 

## Case presentation

A 35-year-old female patient with a BMI of 22 presented to the emergency department (ED) with persistent upper back pain persisting for days following a DN therapy session. She had no significant past medical history and was a nonsmoker. Physical examination revealed multiple trigger points in the upper trapezius and rhomboid muscles. 

The patient was initially referred for DN by his primary care physician for chronic back pain. The patient underwent a ‘superficial’ dry needling session targeting the upper trapezius and rhomboid muscles, and the procedure was performed by a certified physiotherapist (completed a series of courses that cover dry needling techniques, safety, and evidence from the American Academy of Manipulative Therapy [AAMT]) in an outpatient clinic setting three days ago. 

Procedural details: the area of dry needling was on the posterior side of the scapula, the right supraspinatus muscle in a sitting position to be specific, so the bony block on the needle was supposed to be the scapula, not a rib, and the therapist documented hitting the bony block of the scapula with a singular 70 mm needle and then pulling back off of that. The therapist had their other hand on the inferior angle and medial border of the scapula.

The patient did not immediately complain of chest pain or any other breathing issues following the needling procedure. She did report stiffness under the scapula, and the therapist showed the patient a stretch that they could do to get to that area. Following the stretch, the patient then reported a sharp pain underneath the scapula that got worse with inspiration. 

Given the location and nature of the complaint, the therapist looked at this as a muscle strain and proceeded to look at movements that would relieve pain. These maneuvers decreased the symptoms with scapular muscle release. The patient reported an overall decrease in pain with no issues with breathing when she left, and the procedure notes upon review corroborated with the patient's responses post-DN. 

The physical therapist stayed in contact with the patient over the weekend but, due to persistent chest pain, instructed the patient to seek medical care three days after the procedure. 

**Figure 1:**
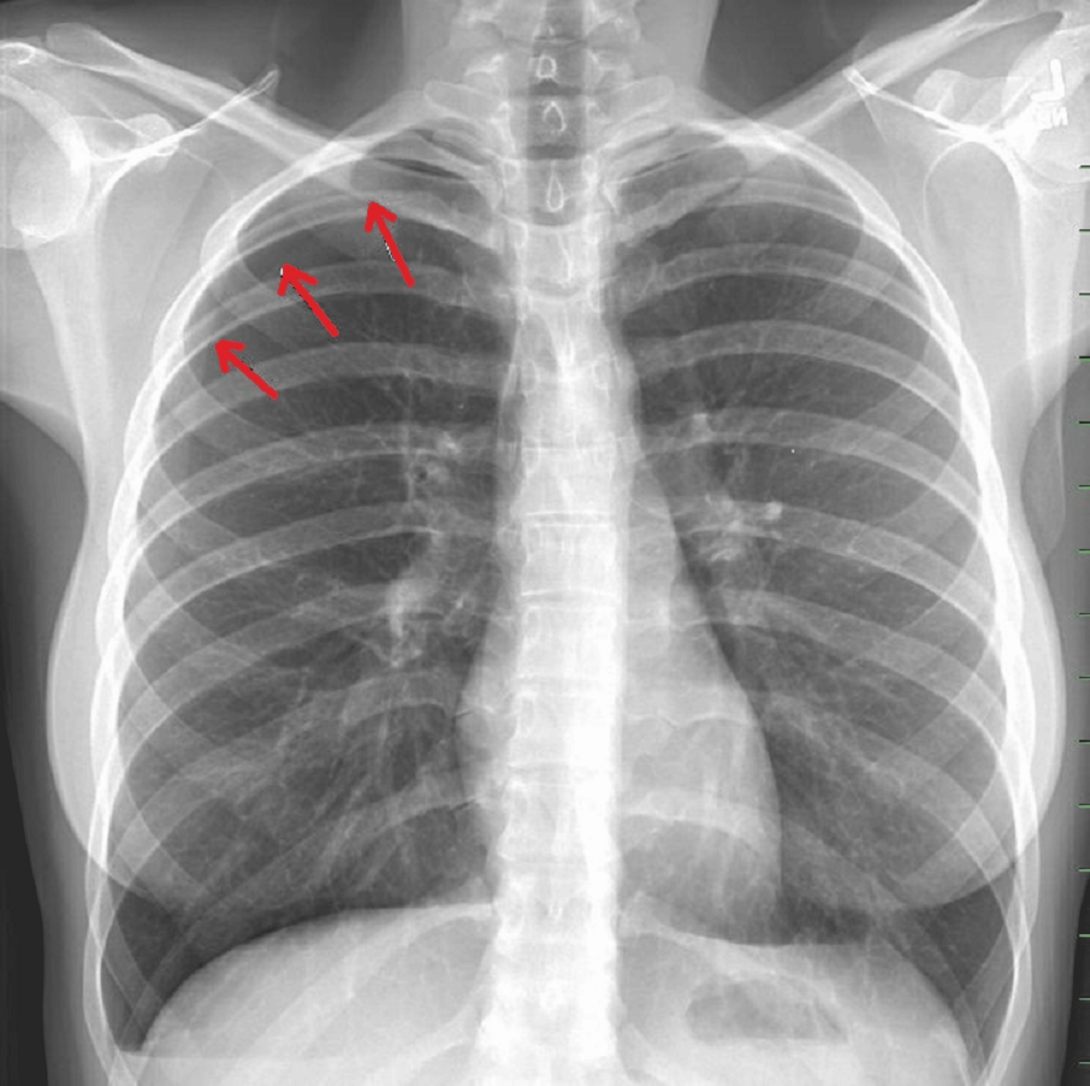
Right upper pneumothorax with collapsed lung border (red arrows).

She presented to the ED due to worsening chest pain with no significant tachypnea or tachycardia, blood pressure of 120/84, and oxygen saturation on air at 96%. Physical examination showed decreased breath sounds on the right side. D-dimer was negative, and a chest X-ray confirmed the presence of a right-sided pneumothorax with 20% lung collapse, and he was diagnosed with iatrogenic pneumothorax secondary to dry needling (Figure 1 shows the collapsed right upper lung border). 

**Figure 2:**
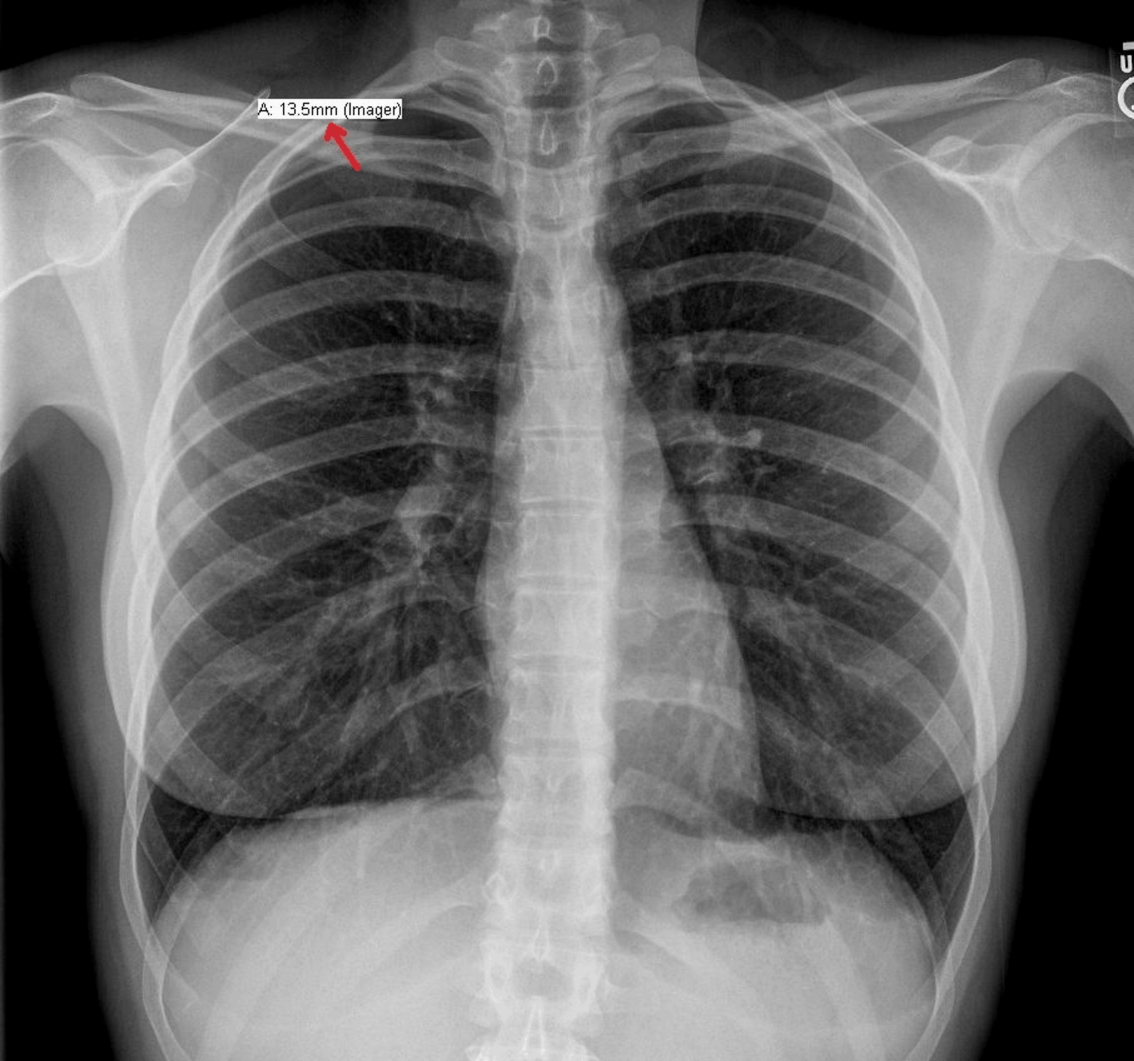
Right upper pneumothorax is improving at 13.5 mm (red arrow).

The patient was managed conservatively with oxygen therapy and close observation in the hospital. Over the next two days, the pneumothorax showed signs of resolution without chest tube placement (Figure 2 shows improvement in the collapsed right upper lung border). The patient was discharged with instructions for follow-up and to avoid similar interventions in the future due to her increased risk related to body habitus and low subcutaneous fat.

## Discussion

Pneumothorax following dry needling can occur if the needle penetrates the pleura, especially when treating areas near the thoracic cavity. The upper trapezius muscle is a high-risk area due to its proximity to the lung apex. When needling around the thoracic region, patients should be warned of the possibility of pneumothorax. Symptoms of pneumothorax may include shortness of breath on exertion, chest pain, dry cough, and decreased breath sounds on auscultation. Because these symptoms may not occur until several hours after the treatment, patients need to be cautioned of this, especially if they are going to be exposed to exercise. If a pneumothorax is suspected, then the patient must be sent urgently to the nearest ED. 

Dry needling training includes both indications and contraindications for the procedures, education, and a competency pass-off with a mentor. In looking back through the education materials from AAMT regarding complications post-DN, documentation included symptoms for pneumothorax like bluish skin, chest pain, difficulty breathing, shortness of breath, coughing, fatigue, etc., which were not immediately observed in this case.

Plausible explanations for pneumothorax in this case are that the scapula was missed the needle hit a rib and surrounding area, and the needle was left in place longer than necessary, leading to migration related to patient movement. It was notable that the ambulatory physiotherapy center was not a busy or dedicated clinic for DN, and the physiotherapists have variable levels of experience with DN. A subsequent peer review of the case suggested that the patient should have been sent to the ED promptly when they had reported 'stiffness' under the scapula post-DN rather than wait for three days.

Pneumothorax cases post-dry needling are rare but well documented. A systematic review indicates an incidence rate of less than 0.01%. Other reported complications include infection, hematoma, and localized pain [4].

The signs and symptoms of pneumothorax may include exertional shortness of breath, dry cough, chest pain, and decreased air entry/breath sounds during auscultation. These symptoms often manifest after the patient has left the clinic and may not appear until several hours following treatment, as highlighted in our case. Patients should be warned about this risk, particularly if they plan to engage in activities involving significant altitude changes, such as flying or scuba diving.

Pneumothorax cases post-DN have mostly been reported when needles are inserted at parasternal or supraclavicular sites, particularly when the placement disregards the fact that the pleura and lung borders extend above the clavicles. Additionally, DN in the infraclavicular and periscapular areas (as in our case) can also lead to pneumothorax. It is worthwhile to note that DN is more site-specific (as compared to acupuncture), based on where a therapist feels a taut band or trigger point.

DN needles are thin, solid monofilament needles similar to acupuncture needles (acupuncture needles are typically 26 to 40 gauge and 0.5 to 2.5 inches long, while dry needling needles can range from 0.16 mm x 25 mm to 0.30 mm x 60 mm), but are very thin; the length of time the needles stay in during dry needling depends on the type of pain being treated. For simple relief, needles may only be inserted for a few seconds, while deeper pain may require 10-15 minutes. 

The largest (60 mm) and the longest needles (30-70 mm) are the needles used for the thickest areas of the body, such as the buttocks and shoulder muscles, as compared to 3-10 mm long needles used for superficial treatments. Acupuncture needles are smoother while dry-needling needles are rougher to better grip muscle tissue. 

Dry needling is increasingly recognized for its therapeutic benefits in treating musculoskeletal pain and dysfunction. The first benefit is pain reduction. Dry needling has shown significant efficacy in reducing pain, particularly in patients with myofascial pain syndrome. Studies suggest that dry needling can lead to immediate and sustained pain relief by deactivating myofascial trigger points, which are hyperirritable spots within a muscle. A systematic review and meta-analysis revealed that dry needling is highly effective in reducing immediate pain and improving pressure pain thresholds in patients dealing with musculoskeletal pain [5]. A 2015 study demonstrated that dry needling significantly reduced pain in patients with chronic tension-type headaches compared to sham needling [6]. 

Mohammad Reza Pour Ahmadi et al. reported that dry needling is highly effective in reducing pain for patients in the short term in patients with tension-type headaches and can significantly improve headache frequency and health-related quality of life [6]. 

Another major benefit is improved function and range of motion. By alleviating muscle tightness and trigger points, dry needling can improve the range of motion and overall function [7]. This is particularly beneficial for athletes and individuals recovering from injuries. Studies have found that dry needling was highly effective in patients undergoing neck dissection, which saw an improvement in the ROM (Range of Motion) and VAS (Visual Analog Scale) score, and dry needling improved functional outcomes in patients with chronic neck pain, enhancing their ability to perform daily activities.

A third benefit of dry needling is enhanced rehabilitation outcomes. Integrating dry needling into physical therapy can expedite recovery and improve rehabilitation outcomes for various musculoskeletal conditions. Randomized controlled trials reported that patients with chronic low back pain who received dry needling alongside conventional physical therapy showed greater improvements in pain and disability scores compared to those receiving physical therapy alone. Research has indicated that dry needling combined with exercise therapy accelerated recovery in patients with patellofemoral pain syndrome. 

Another benefit is the treatment of chronic conditions. Dry needling has been highly effective in managing chronic conditions such as fibromyalgia, chronic tension-type headaches, and neck symptoms. It helps by reducing central sensitization and decreasing the widespread pain associated with these conditions. A study by Adelaida Maria Castro-Sanchez et al. explained that dry needling reduces the intense pain of patients with fibromyalgia syndrome [8]. 

Finally, DN is cost-effective and results in minimal side effects. Compared to other invasive procedures, dry needling is cost-effective and has minimal side effects when performed by trained professionals. The most common side effects are mild and include soreness and minor bruising at the needle site. A cost-effectiveness analysis by Marjolein et al. [9] highlighted that dry needling is a cost-effective intervention for chronic low back pain, offering significant pain relief with minimal adverse effects. Table 1 lists adverse effects as listed by the Health Quality Council of Alberta [10].

**Table 1:** Adverse effects as listed on the Health Quality Council of Alberta, the drawback of these denominators is that they use ALL acupuncture sessions and not the ones with needles over the thorax

Prospective study	#treatments	Minor adverse outcome	Significant adverse outcome	Serious adverse outcome
White et al., 2001 [13]	31822 patients	2135	43	0
MacPherson et al., 2001 [14]	34407 patients	10920	43	0
Melchart et al., 2004 [15]	760000 treatments (97733 patients)	6936	-	6 (2 cases of Pneumothorax)
Witt et al., 2009 [16]	2.2 million treatment (229,230 patients)	19726	4963	-
Brady et al., 2013 [17]	7629 treatments	1463	0	-
Total	3033858 treatments	41180	-	4 cases of pneumothorax

Both the Irish Guidelines (2012) [11] and the Australian Guidelines (2013) [12] emphasize that, even in the absence of international standards, training in dry needling must focus on developing the competency necessary for safe practice.

Emphasis should be placed on distinguishing pain caused by the needling procedure from pain resulting from pleural penetration and potential pneumothorax, which is essential. Pain from pleural puncture is typically described as intense and sharp, often radiating to the shoulder, neck, and occasionally the scapula. It is recommended that standardized processes for healthcare professionals practicing dry needling should be in place to acutely manage and report patient‐related serious adverse outcomes (Table 2).

**Table 2:** Commonly reported risk factors and suggested prevention strategies Table credits: Alok Arora

Risk Factors:
Inadequate anatomical knowledge
Inexperienced practitioner
Deep needle insertion
Thin body habitus of the patient
Prevention:
Thorough anatomical training for practitioners
Use of imaging guidance in high-risk areas
Patient education on the signs and symptoms of pneumothorax
Careful patient selection and consideration of alternative treatments in high-risk individuals

## Conclusions

Dry needling offers substantial benefits for immediate pain relief, improved function, enhanced rehabilitation, and management of chronic conditions. Its efficacy is supported by numerous studies, making it a valuable tool in the treatment of musculoskeletal disorders. While dry needling is an effective treatment modality for musculoskeletal pain, medical professionals and patients must be aware of its potential complications. Proper training, careful technique, and patient monitoring are essential to minimize risks. Early recognition and management of complications like pneumothorax are crucial to prevent severe outcomes; emphasis is needed on recognizing the signs, symptoms, and variability in clinical presentation, which could be different from the classical description. The implementation of preventative measures can aid in mitigating risks. It is crucial that dry needling is performed by trained and certified practitioners to maximize benefits for patients. A balanced approach that weighs the benefits against the potential risks will help maintain the therapeutic efficacy of dry needling while safeguarding patient health. 
